# An Assessment of the Role of Wages and Fringe Benefits on Nurse Aide Turnover in Nursing Homes

**DOI:** 10.1093/geroni/igaf122.1833

**Published:** 2025-12-31

**Authors:** Christopher Brunt, John Bowblis, Robert Applebaum

**Affiliations:** Georgia Southern University, Statesboro, Georgia, United States; Miami University, Oxford, Ohio, United States; Miami University, Oxford, Ohio, United States

## Abstract

Nurse aide (NA) turnover remains a persistent challenge with significant implications for care quality in nursing homes. While wages have been cited as a primary factor influencing turnover, limited research has examined the role of total compensation, including fringe benefits. This study evaluates the relationship between NA turnover and compensation as measured through wages, fringe benefits, and the specific fringe benefits offered across different nursing home ownership types (for-profit, not-for-profit, and government-run). Utilizing national data from 2022 and 2023, we construct a comprehensive dataset from the Centers for Medicare & Medicaid Services’ (CMS) Medicare Cost Reports, Payroll-Based Journal files, and Care Compare archives. We employ regression models controlling for facility and market characteristics, including ownership, payer mix, staffing levels, and state-fixed effects. The results indicate that while higher wages modestly reduce NA turnover, investment in fringe benefits is significantly more effective. While a 10% increase in NA wages reduces turnover by 0.34-0.36 percentage points, a 10% increase in fringe benefit generosity is associated with a 3.5-3.6 percentage point reduction in turnover, with for-profit facilities experiencing even greater effects. Cost-effectiveness analyses indicate that investing the same dollar amount in fringe benefits yields three to sixteen times greater reductions in turnover than an equivalent investment in wages. Specific fringe benefits, such as daycare assistance and tuition reimbursement, further mitigate turnover, while other fringe benefits such as life insurance) are not associated with turnover. These results suggest that policymakers and nursing home administrators seeking to improve workforce stability should prioritize comprehensive compensation strategies.

